# Force-Mediating Magnetic Nanoparticles to Engineer Neuronal Cell Function

**DOI:** 10.3389/fnins.2018.00299

**Published:** 2018-05-15

**Authors:** Trevor J. Gahl, Anja Kunze

**Affiliations:** Department of Electrical and Computer Engineering, Montana State University, Bozeman, MT, United States

**Keywords:** intracellular forces, nanomagnetics, nanoparticles, neurons, cell guidance, cell communication, cell polarity

## Abstract

Cellular processes like membrane deformation, cell migration, and transport of organelles are sensitive to mechanical forces. Technically, these cellular processes can be manipulated through operating forces at a spatial precision in the range of nanometers up to a few micrometers through chaperoning force-mediating nanoparticles in electrical, magnetic, or optical field gradients. But which force-mediating tool is more suitable to manipulate cell migration, and which, to manipulate cell signaling? We review here the differences in forces sensation to control and engineer cellular processes inside and outside the cell, with a special focus on neuronal cells. In addition, we discuss technical details and limitations of different force-mediating approaches and highlight recent advancements of nanomagnetics in cell organization, communication, signaling, and intracellular trafficking. Finally, we give suggestions about how force-mediating nanoparticles can be used to our advantage in next-generation neurotherapeutic devices.

## Key sentence

Quantitative intracellular force interrogation is needed to push the field of neuro mechanobiology into the next level.

## Introduction

Forces inside a cell, called intracellular forces, play an important role during the regulation of a wide range of cellular processes like membrane protrusion, (Ji et al., 2008) cell migration and spreading, (Galbraith and Sheetz, 1998; Pita-Thomas et al., 2015), or transport of intracellular organelles (Svoboda and Block, 1994; Visscher et al., 1999; Klumpp and Lipowsky, 2005; Kunze et al., 2017). These forces can be generated by the cell itself or be imposed on the cell through a force-mediating object or changes in the extracellular environment. The force-mediating object can manipulate cell structures inside the cytosol or outside of the cell at the cell membrane. We will refer here to forces being operated outside of the cell as extracellular forces. In both cases forces can promote or block healthy cell function depending on the magnitude, the direction, the duration, the rate, and the frequency of application.

In the brain, forces are widely associated with traumatic brain injury, where a physical change in the extracellular environment imposes sheer on the brain cells that can lead into damages at the neuronal cell network (Bigler, 2001; Matthew Hemphill et al., 2015), or induce inflammatory neurodegenerative signals (Maneshi et al., 2014). Recent technical advances in capturing mechanical aspects of brain cells in culture have revealed insights into different magnitudes of forces and their impact on regulating brain cell function like calcium signaling (Calabrese et al., 2002; Tay et al., 2016a; Tay and Di Carlo, 2017), neurite elongation (Bray, 1984; Kunze et al., 2015; Pita-Thomas et al., 2015), or vesicle movement (Ahmed et al., 2012; Kunze et al., 2017). These force-mediated cell functions let us hypothesize that forces in the brain may not only cause lesions, far more, they may be used to our advantage in next-generation neurotherapeutic devices.

To integrate force stimulation into therapeutics or diagnostics, however, comes with challenges. How should we design next-generation therapeutic devices to stimulate deep brain tissues without inducing undesired cell effects through high-magnitude forces in more superficial brain tissues? To be able to answer this question, a deeper understanding of the force range affecting single brain cell function and promoting healthy cell communication without generating unintended side effects is required. Furthermore, force-mediated stimulation of cell signals can trigger a variety of intracellular and intercellular downstream processes, inside the stimulated cell, but also on surrounding cell neighbors and tissues, and needs to be better understood for therapeutic applications.

The purpose of this review is to (i) provide an overview about forces at the subcellular scale, (ii) discuss how they can be used to interfere with mammalian cell function, (iii) highlight recent technical advances that allow us to manipulate and interfere with intracellular forces, and (iv) show what needs to be done to advance nanomagnetic force stimulation into a clinical setting. First, we will discuss the variety of force-mediated cellular responses which has been poorly-linked to specific defined magnitudes of forces. For instance, it remains unclear how much force is needed to displace a whole cell body and does the magnitude of force correlate with cell size during migration. Since the magnitude of force is not the only parameter impacting cell function; rate of change, duration, and frequency of force application should also be considered. In a variety of studies, however, the magnitude of force is the only reported or considered parameter. Thus, our review will elaborate on different magnitude ranges of forces used to impact cell signaling, function, communication, and morphology. Furthermore, we will discuss these magnitude ranges specifically for brain cells and provide an overview of force-mediated changes in neuronal cell function. Second, this review we will put a special focus on force-delivery through magnetic nanoparticles which we call nanomagnetic forces. These nanomagnetic forces are mechanical forces induced through a magnetic field gradient on nanometer-sized magnetic particles. Independently on the magnetization properties (ferromagnetic, superparamagnetic), or the magnetic materials of the particle core (magnetite, hematite, migmatite, iron oxide) or the coating materials (silica, dextran), or the functional groups (chitosan, starch, amines, antibodies) a nanomagnetic force is considered as a force acting at the subcellular level within nanometer dimensions. Nanomagnetic forces can be operated inside and outside of cells depending on their geometric and chemical surface properties (Calatayud et al., 2014; Tay et al., 2016a). Because of recent technical advancements, we can operate nanomagnetic forces in parallel through arrays of high magnetic field gradients and apply them to thousands of cells at the same time (Tseng et al., 2012; Kunze et al., 2015; Murray et al., 2016). Third, we will state our opinion about what needs to be done to translate nanomagnetic force stimulation into next-generation neurotherapeutic devices.

## The force-mediating toolbox

**Figure 1:**
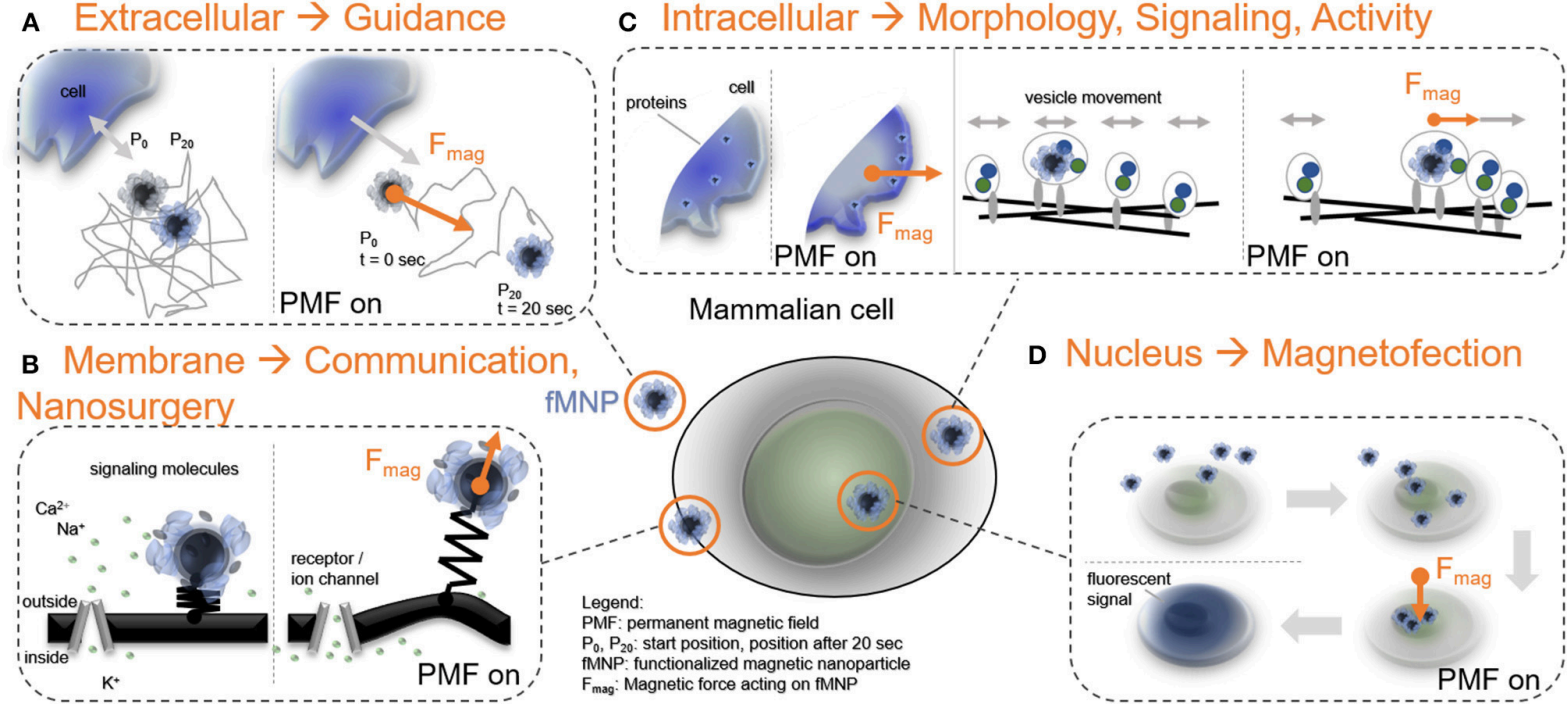
Force-mediating nanoparticles and their interplay with mammalian cell function. **(A)** FMNPs as mediators to direct cell guidance through the extracellular space when exposed to a permanent magnetic field gradient (t: time). **(B)** FMNPs associated to the cell membrane can control receptor functionality, stimulate cell communication, or perform local cell surgeries. **(C)** Positioning of fMNPs inside cells can establish protein gradients and modulate vesicle dynamics. **(D)** Localizing FMNPs to the cell nucleus is utilized to genetically modify cells, here demonstrated through the translation of a fluorescent protein (e.g., GFP, or eGFP).

Forces may act on a mammalian cell or can be generated by a cell. In both cases, forces affect the extracellular or the intracellular environment. To capture and manipulate extracellular or intracellular forces, an object needs to bind, or to enter the mammalian cell to translate a pulling or pushing force on the cellular structure (Figure 1). Dimensions of this object should be chosen in the sub micrometer range to gain high subcellular precision. Thus, most force-related cell applications employ functionalized nanoparticles which is the first tool in the force-mediating toolbox. The second tool is a probe generating a field gradient which imposes a force on the particle and allows the user to control force parameters like direction, magnitude, or frequency.

**Figure 2:**
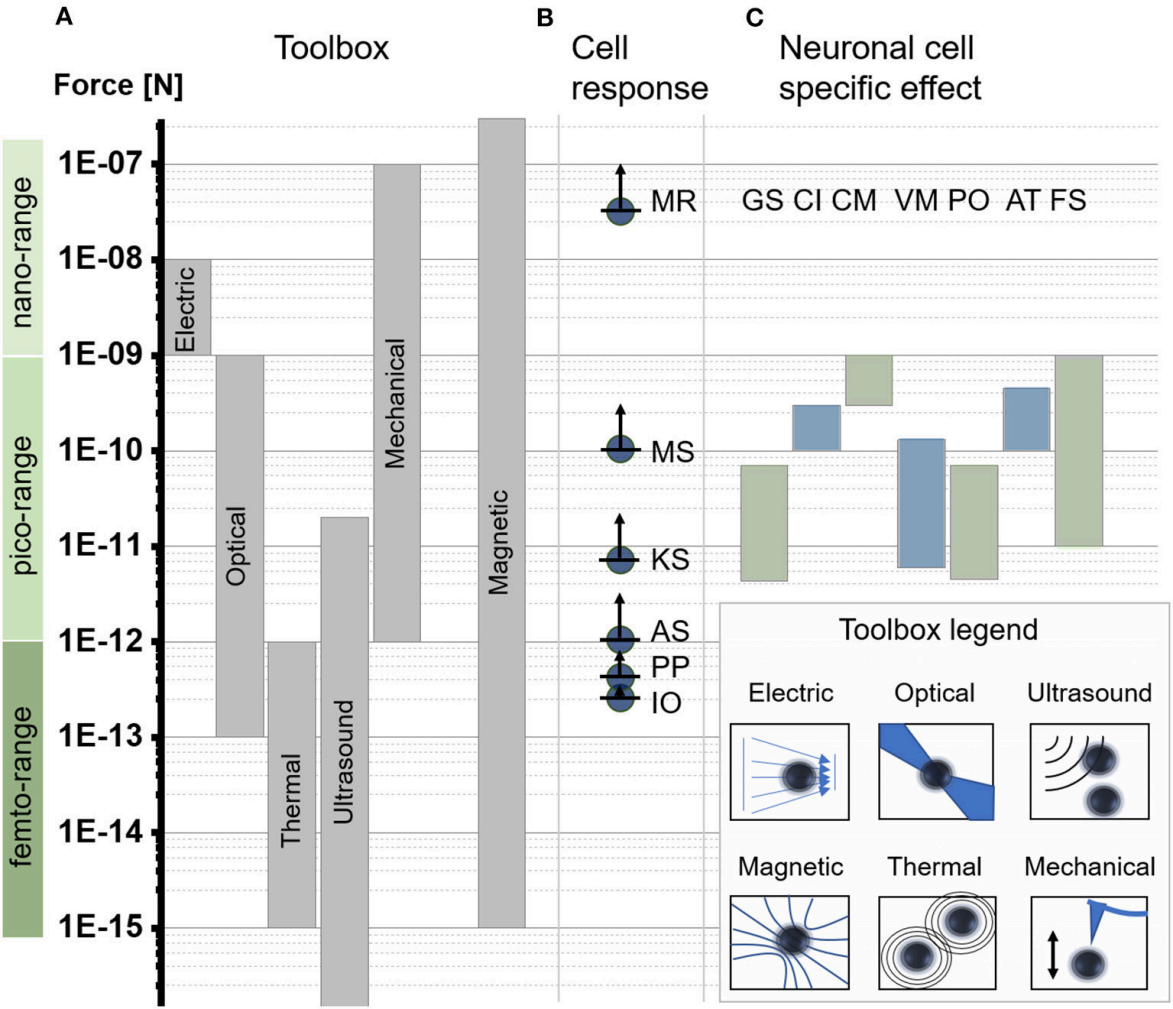
Force scales relevant to subcellular applications. **(A)** Force-generating toolbox to manipulate subcellular forces. Force direction and amplitude are controlled through physical concepts based on electric field gradients = electric, optical tweezers = optical, acoustic tweezers = ultrasound, magnetic tweezers = magnetic, thermal tweezers = thermal, or mechanical actuation = mechanical. (Jeney et al., 2004; Neuman and Nagy, 2008; Mosconi et al., 2011). **(B)** Minimum reported force values required for different cell effects based on computational or experimental models including membrane rupture (MR), (Almeida and Vaz, 1995) microtubules stretching (MS), (Dogterom and Yurke, 1997; Brangwynne, 2006) kinesin motor stalling (KS), (Svoboda and Block, 1994; Visscher et al., 1999) actin stalling (AS), (Footer et al., 2007) protein polymerization (PP), (Footer et al., 2007) ion channel opening and thermal fluctuation (IO). (Meister, 2016) **(C)** Overview of experimentally reported force ranges indicating significant changes in neuronal cell function and morphology induced through a force stimulus. The list highlights general reported mechanical sensitivity for neurons (GS), (Zablotskii et al., 2016a,b) force mediated calcium induction (CI), (Calabrese et al., 2002; Matthews et al., 2010; Maneshi et al., 2014; Tay et al., 2016a) force mediated cell migration/displacement (CM), (Kunze et al., 2015) force mediated modulation of vesicle motion (VM), (Kunze et al., 2017) force mediated protein positioning (PO), (Kunze et al., 2015) force mediated axon towing and stretching (AT), (Bray, 1984; Suter and Miller, 2011) and force mediated filopodia/growth cone stretching (FS). (Franze, 2013).

Technically, we can quantify forces exerted by a cell through traction force microscopy, (Sniadecki et al., 2007; Style et al., 2014; Kilinc et al., 2015), atomic force microscopy (Baumgartner et al., 2003; Elkin et al., 2007; Kuznetsova et al., 2007; Kirmizis and Logothetidis, 2010; Azeloglu and Costa, 2011), or laser ablation (Campàs, 2016). These methods capture changes in cell shape formation, force dynamics of filopodia at growth cones, at the terminal end of neurites, or reveal shootin1–cortactin interactions within the promotion of traction forces at growth cones at high subcellular precision in single cells (Chan and Odde, 2008; Kubo et al., 2015). These methods, however, are not capable to mediate force stimulation. If it is desired to control and operate magnitude and direction of forces at cells, negative pressure or shear stress can be applied on the cell membrane through microchannels, micropipettes, or micro indenters (Fass and Odde, 2003; Huang et al., 2004; Franze, 2013; Campàs, 2016). The dimensions of the channel and micropipette are the determining factor for precision. Alternatively, optical, magnetic, thermal, or electric tweezers are tools that allow for direct force manipulation depending on the physical properties of the force-mediating object (Thoumine et al., 2000; Baumgartner et al., 2003; Jeney et al., 2004; Neuman and Nagy, 2008; Kilinc et al., 2015; Allen Liu, 2016; Tay et al., 2016b; Timonen and Grzybowski, 2017). An external magnetic, optical, or electrical field is required to direct and accelerate the internalized object (Figure 2A). While optical and electrical fields may impact other cell processes, only magnetic fields are transparent to cells. Although, magnetic field gradients for nanomagnetic force manipulation based on permanent magnetic fields (PMF) in combination with micron-sized magnets, or electromagnetic alternating magnetic fields (AMF) are tough to design, they are often the preferred force-mediating toolbox. Lately, technical advancements targeted the cell-by-cell time-consuming data acquisition of magnetic tweezer. The advancement came through microfabricating up to 10,000 parallelized arrays of magnetic field gradient. The fabrication approach based on permalloy, borrowed from solid state devices, was integrated into cell culture chips of the size of few millimeters (Tseng et al., 2012; Kunze et al., 2015, 2017; Murray et al., 2016; Tay et al., 2016a). Acoustic tweezers are also used to move and modulate intracellular trafficking. Standing acoustic field can be created using ultrasonic waves which causes the objects to feel acoustic radiation force. This force is used to move objects to acoustic pressure nodes and antinodes (Chen et al., 2014). This method has advantages over optical tweezers such as causing less damage to organelles while applying more force.

In the case of an extracellular force stimulus, the force-mediating object can either work as a mechanical cue (Figure 1A), or as a membrane actuator (Figure 1B), or actively target transmembrane proteins. Distinct force applications at the cell membrane can be achieved through selective surface coatings on the nanoparticle (Calatayud et al., 2014; de Castro et al., 2018). Through forces acting on the cell membrane, cell protrusion can be initiated to start growing an axon in neurons (Fass and Odde, 2003; Ji et al., 2008; Betz et al., 2011; Diz-Muñoz et al., 2013; Pita-Thomas et al., 2015; Bidan et al., 2018). Extracellular forces at higher magnitudes can elongate neurites or growth cones (Bray, 1984; Zheng, 1991; Suter and Miller, 2011; Kilinc et al., 2015; Ren et al., 2018), guide cell displacement and migration, (Kunze et al., 2015; Doolin and Stroka, 2018; Van Helvert et al., 2018) or open membrane channels to interfere with neuronal cell communication (McBride and Hamill, 1993; Martinac, 2004; Morris and Juranka, 2007; Reeves et al., 2008; Beyder, 2010; Sanjeev Ranade et al., 2015; Tay et al., 2016a).

When it comes to the intracellular space, forces are involved in molecular motor transport (Svoboda and Block, 1994; Visscher et al., 1999; Klumpp and Lipowsky, 2005), the formation of cytoskeletal structures like actin filaments and microtubules (Dogterom and Yurke, 1997; Brangwynne, 2006) and the local signaling of proteins (Figure 1C) (Kosztin et al., 2002). To study forces involved in the intracellular space with high precision, we need the force-mediating object to enter the cell through phago-, pino-, or endocytosis. The uptake mechanism of the force-mediating object highly depends on the cell type, the metabolic state of the cell and particle properties like shape, size and surface functionality (Lesniak et al., 2012; Tay et al., 2016c; Suarato et al., 2017). From previous studies with optical tweezers, we know that single molecular motor and cytoskeleton filament forces are within the lower pico-Newton range, e.g., kinesin motors stall between five to six pico-Newtons (Svoboda and Block, 1994; Visscher et al., 1999). The ability to precisely operate intracellular forces, however, is still a challenge due to low experimental through-put and limited targeting specificity of the force-mediating object inside mammalian cells. Future work is required to systematically generate a map of force ranges considering magnitudes, duration and rate of application in relation with cell specific effects to precisely specify force sensitivity in brain cells.

## Neuronal cell force sensitivity

To mechanically engineer cellular effects, it is essential to know the exact force range for (a) the desired cell effect and (b) the different force-mediating tools. Based on theoretical and empirical observations (Figure 2B), cells are sensitive over three distinct ranges of force magnitude (Footer et al., 2007; Kenry and Lim, 2016; Meister, 2016; Zablotskii et al., 2016a). The three force ranges can be separated into nano-Newton forces (1–1,000 nN), pico-Newton forces (1–1,000 pN), and femto-Newton forces (1–1,000 fN). Within these force ranges a single event at the subcellular space can be the opening of a calcium channel. An experimentally-based smallest magnitude of 200 fN has been reported to open a force-sensitive TREK-1 ion channel with a 250 nm-diameter particle in auditory hair cells, or kidney fibroblast-like cells (Howard and Hudspeth, 1988; Hughes et al., 2008; Meister, 2016). Magnitude of forces between 150 pN and 5 nN acting on mechanosensitive ion channels, via integrin-cytoskeleton coupling using a 4.5 μm-diameter particle, triggered calcium influx in endothelial cells (Matthews et al., 2006, 2010). Mechanical sensitive calcium channels can also be found in primary neurons, where significant increases in calcium influx were observed for neurons similar to endothelial cells above 150 pN. (Matthews et al., 2006; Tay et al., 2016a; Tay and Di Carlo, 2017). The gap between the reported femto- and pico-Newton magnitude of forces to stimulate calcium influx can be attributed to the difference between a delivering vs. acting force. While a larger particle can deliver a higher force to the cell, or specifically to the integrins, or to the ion channels, it also can act on or be targeted to a higher number of ion channels at a cell surface than a smaller particle. The actual minimal amount of force required to stimulate the opening of an ion channel remains then similar. This observation suggests that cells which are exposed to higher magnitude or rate of forces should show a stronger cellular effect. For the calcium channel opening and protein displacement this effect has been demonstrated within a distinct force interval (Kunze et al., 2015; Tay and Di Carlo, 2017). Observations of neuronal cell behavior across a larger range of magnitudes of forces, however, have revealed that above certain force thresholds cells change their entire response. While forces in the lower pico-Newton range interfere with cell functioning; forces in the higher pico-Newton range may induce cell transformative effects that impact the cell morphology or may break through the cell membrane.

Figure 2B highlights the most important minimum cellular force thresholds above which major changes in cellular responses were reported. In the upper femto-Newton range, 200 fN are necessary to open a single ion channel in a cellular membrane and to overcome thermal fluctuation effects (Howard and Hudspeth, 1988; Dobson, 2008; Hughes et al., 2008). At least 200 fN to 500 fN have been reported to induce actin polymerization leading to a minimal stalling force threshold for actin polymerization of 1 pN (Tyler, 2012). To stop the motion of a single kinesin motor, 5.6 pN are required (Svoboda and Block, 1994; Visscher et al., 1999). In the same range, at 5 pN, stalling forces for single microtubules have been reported (Tyler, 2012). Increasing intracellular forces from the lower to the higher pico-Newton range show a different picture. Bundles of microtubules in combination with microtubules-associated proteins can withstand up 100 pN (Tyler, 2012). Above this threshold the cell morphology starts to change. Subcellular structures like microtubules and lipid membranes appear to destabilize and to transform into a fluidic state allowing the cell to deform their cellular membrane without rupturing it (Diz-Muñoz et al., 2013; Pita-Thomas et al., 2015). The next reported force threshold occurs in the nano-Newton range, around 25 nN, which is the maximum force a cell membrane can withstand before it ruptures (Almeida and Vaz, 1995). The rupture, however, can be very local and may be reversible. In this case live cell nanosurgery becomes possible (Obataya et al., 2005; Praveenkumar et al., 2015). The list of reviewed force thresholds has its limits when it comes to spatio-temporal changes. A lower force threshold might be possible for certain cell effects when applied just long enough, or when operated faster than currently possible.

Focusing on brain cells the force sensitivity range is much more limited. Although most cell-generic force thresholds apply for neurons, force-mediated cellular effects are only reported in the pico-Newton range (Figure 2C). Operating and controlling forces across the whole cell sensitivity range, quickly limits our toolbox to magnetic tweezers and systems based on the current technical state of art. The specific neuronal cell sensitivity, however, promotes optical and magnetic tweezers.

## Organizing cell tissue constructs with nano-guides

Replicating the filigree organization of biological tissues has been the focus of many studies during the last 20 years (Butler et al., 2000; Goldberg et al., 2007; Pampaloni et al., 2007; Sakar and Baker, 2018). Biological tissues are highly organized constructs which consist of a diverse range of cell types, which are assembled into layers of heterogenous cell densities to perform different function. Integrated in the constructs is a densely branched vascular system that provides oxygen and nutrition.

**Figure 3:**
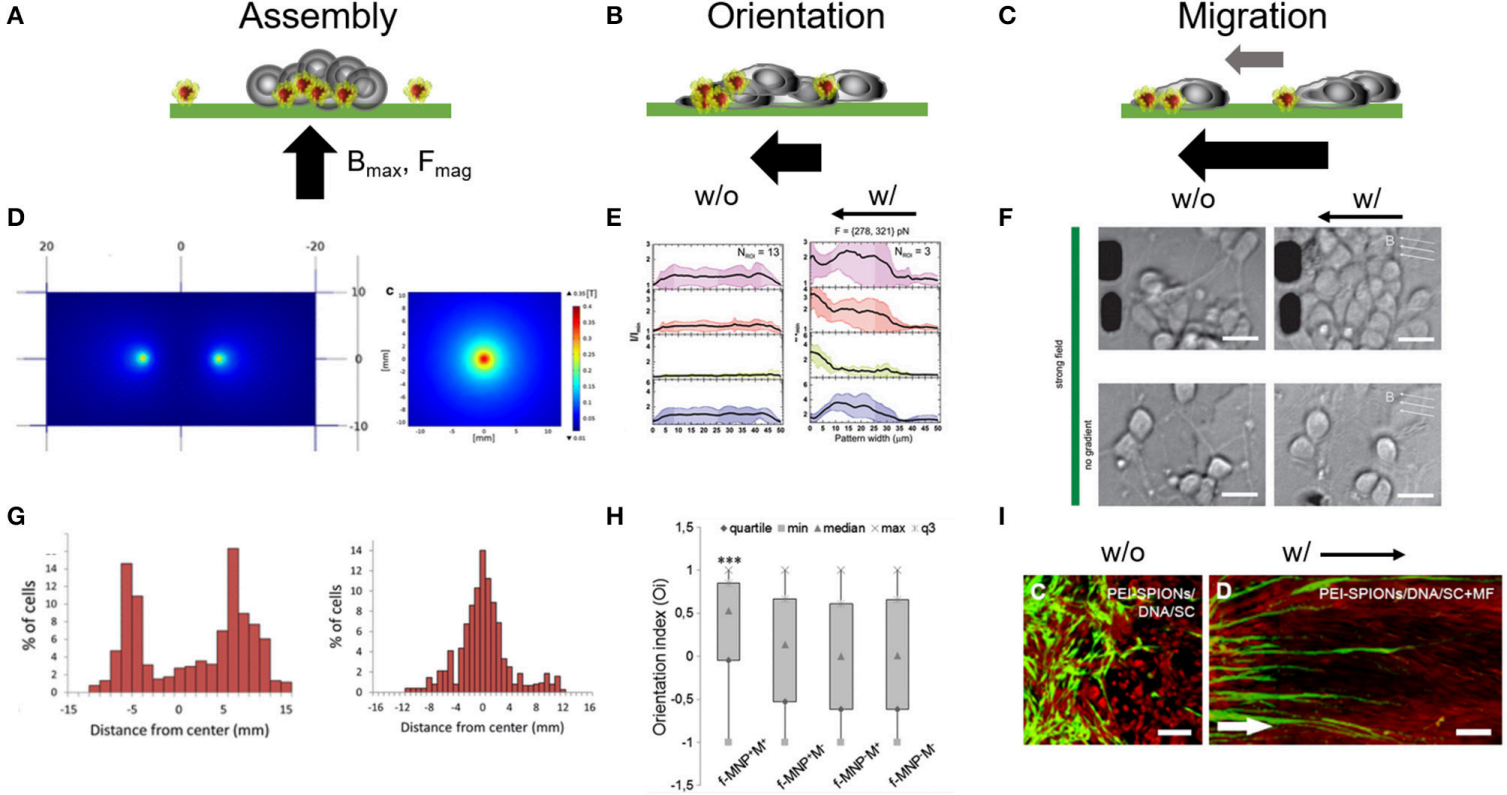
Magnetic forces as cell patterning mediators. Tuning magnetic force amplitudes (F_mag_), the position of the maximal magnetic field (B_max_) and the orientation of the magnetic field pole indicated through the black arrow provides a versatile approach for cell assembly. **(A–C)** Schematic represents different magnetic field gradient orientations and magnitudes and its impact on cell assembly and organization. **(D)** Single vs. two pole magnetic field gradient spots for positioning of cells. Reproduced with permission from Marcus et al. (2016), Copyright © 2016, BioMed Central. **(E)** Fluorescence distribution plots taken from primary cortical neuron cultures show a shift of intracellular markers toward left oriented magnetic gradient forces. w/o, no magnetic field; w/, with magnetic field. Reproduced with permission from Kunze et al. (2015). **(F)** Primary cortical neurons dissociated from rat brain tissues (E18) were cultured on poly-l-lysine surfaces and exposed to fMNPs after being 24 h in culture. These neurons grow and form neurite networks under magnetic fields and start migration toward magnetic field poles under strong magnetic forces (> 250 pN). Scale bar = 12 μm. Reproduced with permission from Kunze et al. (2015), Copyright © 2015, American Chemical Society. **(G)** Histogram plots of accumulated neuron-like cells which were cultured above the single and two pole patterns, respectively. **(H)** Orientation index extracted from PC12 that were observed to align in parallel to magnetic field orientation after being cultured with fMNPs. f-MNP-M^+^, fMNPs with magnetic field; f-MNP-M^−^, fMNPs without magnetic field. Reproduced with permission from Riggio et al. (2014), Copyright © 2014, Elsevier Inc. **(I)** Schwann cells migrate into astrocyte-rich region under an oriented magnetic field gradient after internalizing PEI-fMNPs (PEI-SPIONs). White arrow indicates direction of magnetic pole. Scale bar = 100 μm. Reproduced with permission from Xia et al. (2016), Copyright © 2016, Dove Medical Press Limited.

Most tissue engineering studies report on manipulating the chemical and mechanical properties of the extracellular environment to trigger a desired cell response, e.g., local cell organization, cell orientation, cell migration, and cell network formation, which then potentially leads to the desired tissue organization and physiology. In contrast to engineering the extracellular environment, direct positioning of cellular bodies allows us to engineer cell tissues from the bottom to the top. In this context, magnetic gradients and forces are utilized to collect and assemble mammalian cells to specific local positions on a plane in a controlled manner (Figure 3A) (Tanase et al., 2005; Ino et al., 2007, 2008; Rampini et al., 2015; Zablotskii et al., 2016a,b). The orientation of the magnetic field poles and the magnitude of the magnetic force are the most determining parameters to either assemble, orient or direct cell position (Figures 3A–C). To attract nonadherent cells to specific places on a surface or in suspension the cell body needs to incorporate magnetic materials or needs to attach to magnetic guides. The position of the magnetic poles then attracts the magnetic guides allowing the cells to locally attach to the surface, to assemble into a tissue, or form multi-layered spheroid clusters in connection with additional cell neighbors and cell layers (Ino et al., 2007; Ito and Kamihira, 2011; Lee E. et al., 2014). Using this concept, Marcus et al. positioned rat pheochromocytoma PC12 cells on multi-pole arrays (Figures 3D,G) (Marcus et al., 2016). When cell bodies already adhered to their growth surface, applying magnetic gradients, and forces can orient the cellular morphology (Figures 3B,E). Specifically, fine tuning the magnetic field gradient forces provides the possibility to orient primary cortical neuron cell growth in the lower pico-newton range (Figure 3E) and to induce cell migration in the higher pico-newton range (Figures 3C,F) (Kunze et al., 2015). PC12 cell orientation was also reported for aligned magnetic nanoparticle guides in a two-pole magnetic field (Figure 3H) (Riggio et al., 2014). Subsequent seeding of cells over the unoccupied cell regions would allow for co-culturing of different cell types cell-by-cell or layer-by-layer. Magnetic gradient forces were also reported to support Schwann cell migration through astrocyte-rich cell regions (Figure 3I) (Xia et al., 2016; Huang et al., 2017). What remains unclear is how easy magnetic-guided tissue engineering can be applied to primary neurons. Above mentioned studies focused on PC12 cells which are neuron-like cells, or non-neuronal brain cells known to show differences in particle endocytosis in comparison to primary neurons (Pinkernelle et al., 2012).

Another important aspect of tissue growth and assembly is the time-varying relation between cell migration and function. Throughout the development of newly forming tissues, the individual cells must adapt to changes in the biochemical and biomechanical environment and decide to leave, stay, or modulate their environment. Little is known about how fast cells respond to biomechanical changes, or what happens if the cell fails to do so. Magnetic field gradients can be switched on and off, either through an external electrical current or through removing the externally applied permanent magnetic field. Combining magnetic field gradients with time-varying cell assays seems to be a versatile way to study the adaption of cell tissue functionality in changing environments. Overall, utilizing magnetic gradients and magnetic forces are an attractive method to assemble and grow cells into complex constructs and to further investigate time-varying changes in the cellular environment and their effect on cell function.

## Modulating cell communication with nanomagnetic forces

**Figure 4:**
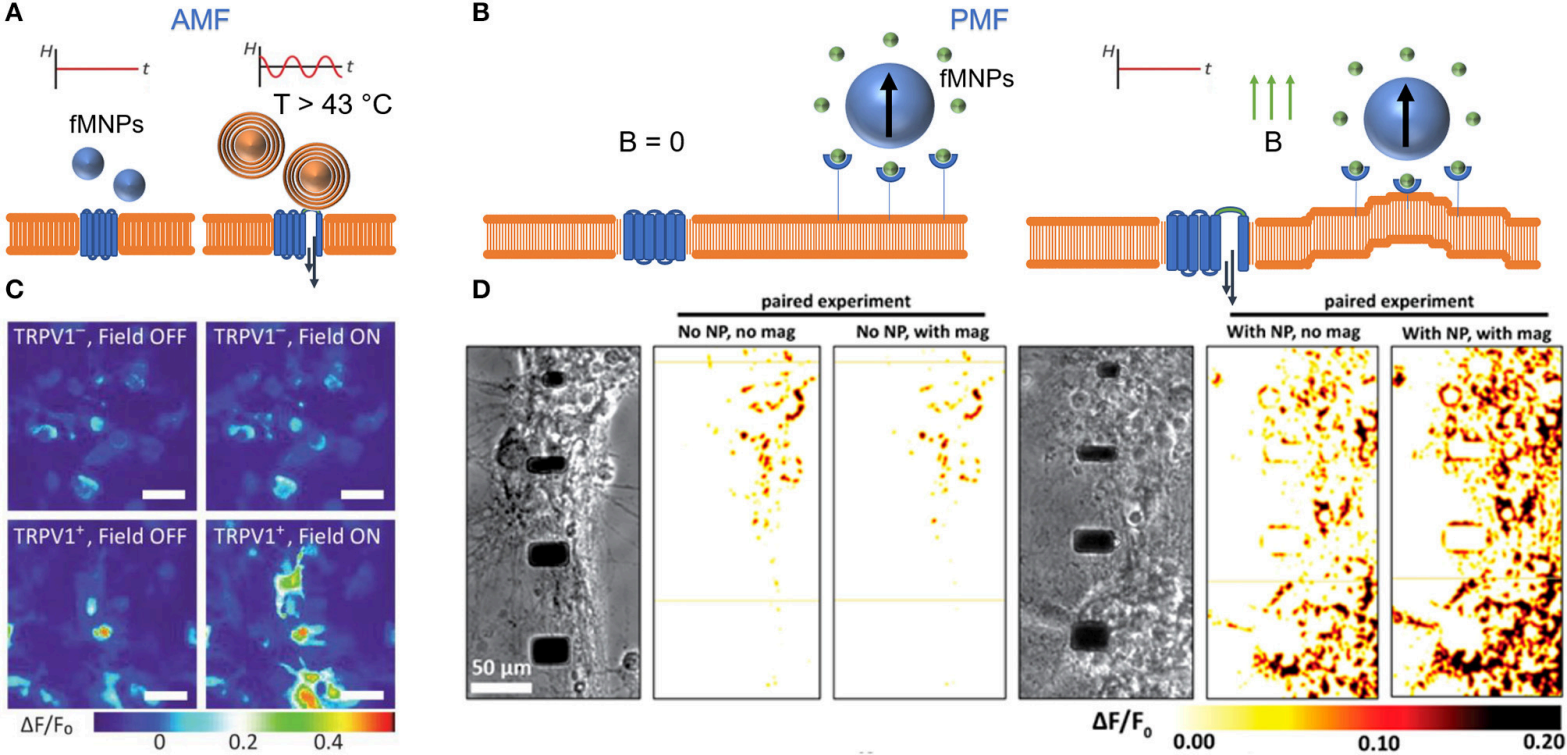
Controlling calcium influx with alternating (AMF) and permanent magnetic fields (PMF). **(A)** Heat stimulation of TRPV1 through fMNP-mediated heat induction in AMF. **(B)** FMNP-mediated calcium channel activation via membrane bending in PMF. “B = 0” indicates no magnetic field. **(C)** False color heat maps show changes in fluorescently-labeled intracellular calcium concentration in TRPV1- and TRPV+ HEK293FT cells before and during AMF stimulation. Scale bar = 50 μm. Reproduce with permission from Chen et al. (2015), Copyright © 2015, American Association for the Advancement of Science. **(D)** False color heat maps show changes in fluorescently-labeled intracellular calcium influx in primary cortical neurons (E18, rat) with and without fMNPs and with and without PMF stimulation. Reproduce with permission from Tay et al. (2016a), Copyright © 2016, American Chemical Society.

Neuronal cells propagate information based on ionic sodium and potassium signals, which can be electrically monitored. Calcium signals play an important role in this ionic signaling and signal propagation mechanism (Rasmussen, 1970). Through precisely activating Ca^2+^ channels, calcium influx can be stimulated, protein function can be post-translationally modified, or gene transcription can be induced (Berridge et al., 1998, 2000; West et al., 2001). Most recently, calcium influx was remotely controlled through heat induction or force manipulation using alternating or permanent magnetic field, respectively (Figure 4; Calabrese et al., 2002; Maneshi et al., 2014; Bonnemay et al., 2015; Tay et al., 2016a; Tay and Di Carlo, 2017). Both approaches have been proven to be beneficial to induce calcium signals in a confined area, or at distinct subcellular compartments. Heat-mediated calcium influx occurs when an alternating magnetic field is applied in conjunction with an overexpression of TRPV+ channels in neurons (Figure 4A), resulting in locally increased calcium concentrations in primary hippocampal neurons (Figure 4B; Chen et al., 2015). However, the effect of sustained heating over more than a few minutes needs to be further demonstrated. Nimpf and Keays provided in this context further critical comments about limitations and reproducibility's of heat mediated magnetogenetic approaches (Nimpf and Keays, 2017).

While modulating cell communication through heat is applicable to the *in vivo* environment of neurons, the host organism must be genetically modified. In contrast to heat induction, mechanical forces can bend the cell membrane and interrogate associated calcium channels (Figure 4B) (Matthews et al., 2006, 2010). Tay et al. demonstrated an average increment of calcium influx by 20 % for magnetic nanoparticles imposing forces above ~200 pN at the cell membrane (Figure 4B) and a 10 % increase for forces operating inside the cell in primary cortical neurons (Tay et al., 2016a; Tay and Di Carlo, 2017). Additionally, Hughes et al. have demonstrated the selective activation of ion channels via magnetic nanoparticles (Hughes et al., 2008). Magnetic nanoparticels were introduced to TREK-1 transfected COS-7 cells and by placing a rare earth magnet ~1.5 cm away from the cells, a magnetic field of ~80 mT was applied with a field gradient of ~5.5 Tm^−1^. The results indicated that channel activation occurred at ~0.2 pN per particle when using 250 nm particles (Hughes et al., 2008). The difference in forces magnitude between the two studies may be due to differences in membrane targeting, or due to differences in the sensitivity of the optical vs. electrophysiological probing method. While Tay et al. used nanomagnetic forces to bend the membrane and to mechanically activate N-type calcium channels, Hughes et al. specifically targeted the magnetic particles in their study to the mechanosensitive TREK-1 ion channel. Alternatively, the magnetic field can also be operated either to induce torque (Hudspeth et al., 2000; Mosconi et al., 2011). or to induce tensile stretch on mammalian cells to stimulate ion channels and cell communication (Lee J. et al., 2014). Recently, the torque approach has been used in conjunction with confocal microscopy to image force responses in living cells (Zhang et al., 2017). The approach has been further expanded upon by Chen et al. through the integration of a multi-pole electromagnet that allows for control of both the twisting direction as well as the magnetic strength (Chen et al., 2016).

While multiple studies have examined the usage of magnetic forces for channel activation *in vitro* translating nanomagnetic force stimulation *in vivo* still needs to be shown and will require accurate operation and positioning of magnetic field gradients in the body. Using magnetic implants based on current chip technology, or electromagnetic micro needles (Matthews et al., 2004) opens the possibility to operate calcium communication inside the brain through mechanical stimuli, however, it will remain an invasive procedure.

## Compartmentalizing intracellular proteins

Separating intracellular organelles and proteins into distinct compartments within a cell is a critical event during cell differentiation, cell mitosis, cell signaling, and to establish functional cell polarity in neurons (Bradke and Dotti, 1997, 2000; Bentley and Banker, 2016; Hansen et al., 2017). Compartmentalizing the location of proteins in the cytosol can be effectively altered though the application of subcellular forces. Mechanically manipulating the position of proteins can be controlled through endocytosed magnetic nanoparticles within magnetic field gradients (Pan et al., 2012; Bonnemay et al., 2013; Etoc et al., 2013, 2015; Kunze et al., 2015; Hughes and Kumar, 2016; Ducasse et al., 2017; Liße et al., 2017; Monzel et al., 2017). The force range to establish a specific protein gradient, however, should leave the tension at the cell membrane at a homeostatic level. This homeostatic level at the cell membrane is a balance between intracellular structural forces and extracellular adhesive forces keeping the cell membrane intact and the cell morphology at a constant shape. Keeping the cell membrane at a homeostatic constant level is highly essential for healthy functioning of cells, tissues, and organs (Smith, 2010). In contrast, impaired homeostatic levels were reported to correlate with cancer cell formation, and dysfunctional cell behavior (Dityatev et al., 2010; Gilbert and Weaver, 2017).

**Figure 5:**
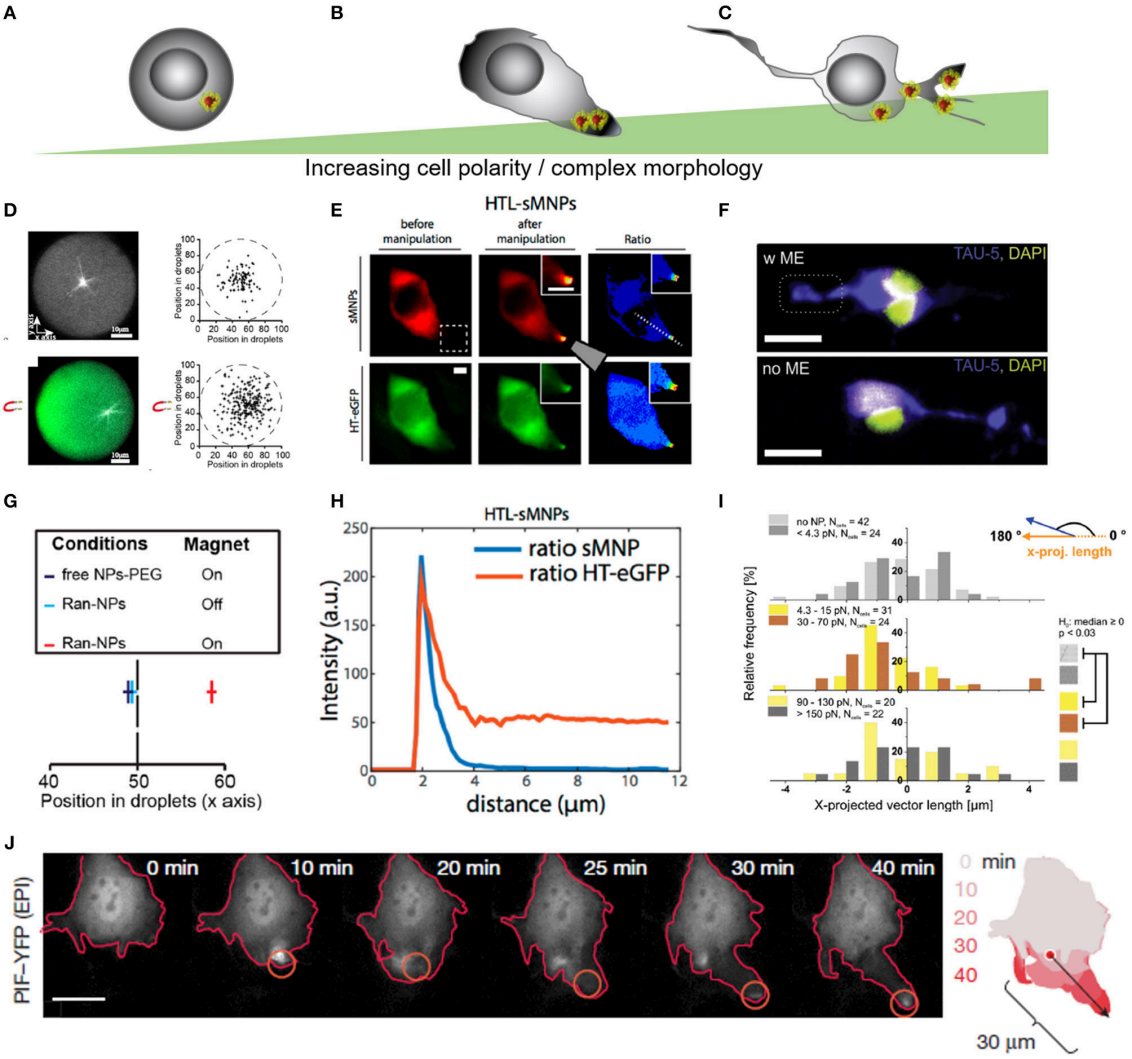
Force-mediated protein sorting inside cells across different levels of complexity in cell morphology. **(A–C)** Schematic representation of different levels of cell complexity ranging from almost perfectly round to highly branched structures. **(D)** Microtubules nucleation in artificial, micro-scaled lipid droplets de-centralize the nucleation zone through the application of magnetic field gradient. Magnetic forces off-center nucleation position of microtubules through repulsion. Forces were estimated in the femtonewton range. **(E)** Altered protein positioning (HaloTag-eGFP) through nanomagnetic forces operated by magnetic tweezers in HeLa cells. Scale bar = 10 μm. HTL-sMNPs, HaloTag-ligand-silica-based magnetic nanoparticles. **(F)** Primary cortical neurons with superparamagnetic nanoparticles re-assemble Tau proteins toward the magnetic field gradient when exposed to a permanent magnetic field. Scale bar = 16 μm. **(G)** Microtubules nucleation position of RanGTP-magnetic nanoparticles (Ran-NPs) without (Off) and with (On) magnetic forces. **(D,G)** Reproduced with permission from Bonnemay et al. (2013), Copyright © 2013, American Chemical Society. **(H)** Surface intensity plot shows correlation between nanoparticles (HTL-sMNPs = sMNPs) and protein assembly (HT-eGFP) in transfected HeLa cells dropping away from the magnetic tip. **(E,H)** Reproduced with permission from Etoc et al. (2015), Copyright © 2015, American Chemical Society. **(I)** Histogram plot for the nanomagnetic force range were protein assembly was significant different from its native distribution. **(F,I)** Adapted from Kunze et al. (2015), Copyright © 2015, American Chemical Society. **(J)** Force-mediated local activation actin cytoskeleton dynamics through dragging Rho-family GTPases proteins. Reproduced with permission from Levskaya et al. (2009), Copyright © 2009, Springer Nature.

Different cell types, however, develop different cell morphologies (Figures 5A–C). While epithelia cells keep their cell membrane uniformly distributed around the nucleus, neurons grow their tangibles heterogeneously and far away from the nucleus, which results in a more complex cell morphology. The resulting level of cell membrane homeostasis may then differ between the different cell types. Applying a localized force stimulus for protein sorting on intracellular compartments will also put the cytoskeleton, the cellular membrane, and protein clusters under tensions. Hence, redistributing proteins based on magnetic or optical gradients in morphologically complex cells might require a higher force gradient, or longer force application than in spherical cells. To avoid damaging the cell membrane, or blocking intracellular transport through narrowed cellular features, nanomagnetic force amplitudes need to be adapted to the homeostatic cell level and should be ideally uniform across the entire cell, which is technically a challenge. Thus, magnetically sorting proteins in cells where cell morphologies ranges from a less to a more complex architecture will require special care regarding the application of force amplitudes.

In spherical cell-like liposomes and *Xenopus laevis* eggs asymmetric spots of microtubule fibers were assembled through positioning RanGTP proteins conjugated to superparamagnetic nanoparticles under the operation of magnetic field gradients (Figures 5D,G) (Bonnemay et al., 2013; Hoffmann et al., 2013; Ducasse et al., 2017). The authors reported operating nanomagnetic forces in the femtonewton range below the thermal fluctuation threshold (Ducasse et al., 2017). In HeLa cells, protein organization was spatially and temporally altered within a range of few femtonewtons up to 30 pNs depending on the magnetic particle size (Figure 5E,H; Etoc et al., 2015). In NIH 3T3 cells, Levskaya et al. regulated the actin cytoskeleton dynamics through local activation of Rho-family GTPases proteins (Figure 5J; Levskaya et al., 2009). The assembly of proteins was enabled through a genetically encoded light-control system which was operated within the pico-Newton range. In rat cortical neurons, cell morphology is more complex and proteins are more polarized than in other mammalian cell types, nanomagnetic forces between 4.3 and 70 pN have been shown to sort Tau proteins around a 180° axis (Figures 5F,I; Kunze et al., 2015). Overall, magnetic forces have been probed to modulate protein gradients across a variety of cell morphologies, what remains unclear is how strong does the cell morphology interferes with the formation of force-mediated protein gradients. The studies, we mentioned, suggest a spectrum of required force ranges for subcellular protein assembly and redistribution that might depend on the complexity of the cellular morphology, but also on particle functionalization and endocytosis pathways. Thus, a variety of investigations are required to better understand how intracellular protein sorting can be linked to cell disease, functionality, growth and death.

## Modulating intracellular traffic

**Figure 6:**
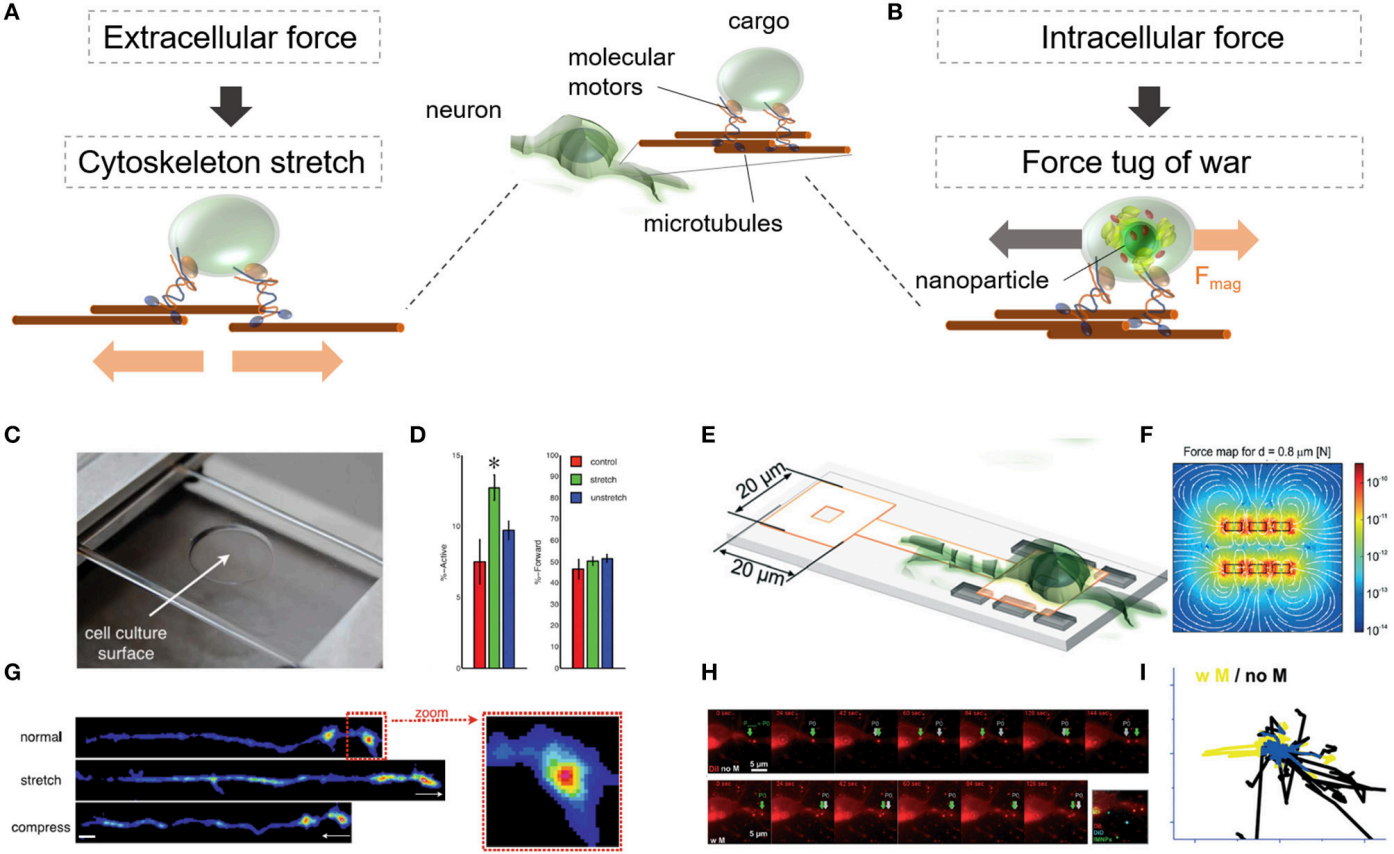
Dynamic behavior of intracellular vesicles is sensitive to force-mediated changes inside and outside of neurons. Changes in vesicle dynamics are studied through **(A)** a stretchable or **(B)** a localized nanomagnetic cell culture platform. **(C)** The stretchable cell culture surface imposes a uniform elongation on adherent parts of the cell body and cytoskeleton. **(D)** Histogram shows differences in force-mediated active vs. axonal forward (anterograde) transport. **(E)** On chip method to mechanically interfere with vesicle dynamics inside neurons through nanomagnetic forces. **(F)** Estimated nanomagnetic force map. **(G)** Stretch mediates the increase of synaptic vesicles in neuromuscular synapses in *Drosophila* embryonic motor neurons. **(H)** Nanoparticle-laden lipid vesicles in primary cortical neurons alter their movement pattern under magnetic forces. **(I)** Extracted vesicle tracks without (no M) and with (w M) nanomagnetic forces. **(C,D,G)** Reproduced with permission from Ahmed et al. (2012,2013), Copyright © 2012, Royal Society of Chemistry. **(E,F,H,I)** were adapted and reproduced with permission from Kunze et al. (2017), Copyright © 2017, Royal Society of Chemistry.

Vesicle dynamics are a key component of transporting molecules inside cells to distinct subcellular sites for proper cell growth, signaling, and maintenance of homeostasis. Perturbating intracellular vesicle dynamics helps us to better understand the role of vesicle transport in a variety of diseases mechanism and propagation. Conventionally, vesicle dynamics were altered through genetically modified signaling pathways, or biochemically inhibiting transport dynamics. A comprehensive review about these approaches is provided by van Bergeijk et al. (2016) A genetically, or chemically independent approach to vesicle transport is through the application of mechanical forces. Two distinct methods employ mechanical tension on transport dynamics of vesicles in neuronal cells (Siechen et al., 2009; Ahmed et al., 2012, 2013; Kunze et al., 2017). The difference between the two methods are depicted in Figure 6. Both methods apply mechanical forces, either through substrate stretching outside of the cell (Figure 6A; Ahmed et al., 2010, 2013) or through nanomagnetic forces inside the cell (Figure 6B; Kunze et al., 2017).

Depending on the application of the extracellular force via a stretchable cell culture platform (Figure 6C), the stretch, or buckle will result in a uniform elongation, or compression of the whole cell body in adherent cells including their cellular compartments. Constantly stretched *in vivo* axons in *Drosophila* embryonic motor neurons (where only the embryonic body was fixed to the platform) accumulated synaptotagmin-labeled vesicles in the axonal tip in the absence of Ca^2+^ (Figure 6G). After the tension was removed, the effect persisted for at least 30 min. Because vesicles are constantly transported forward (anterograde = toward the synapse) and backward (retrograde = away from the synapse), the stretch-mediated effect was reported to be more dominant in the forward then in the backward transport (Figure 6D). Additionally, it was discovered that compressive strain along Drosophila motor neuron axons did not increase synaptic vesicle accumulation and decreased tension in Aplysia neurons which resulted in disrupted motion of large dense core vesicles. This effect persisted in Aplysia neurons for at least 15 min after standard tension was restored (Ahmed et al., 2012). One possible explanation for this effect can be the developmental state of the axonal tip. Although the *in vivo* Drosophila neuron had established a neuromuscular synapse, the *in vitro* Aplysia neurons was in a pre-developmental synaptic state. This finding opens the questions if force-mediated vesicle dynamics highly depends on the developmental state of the cellular compartment and its subcellular cytosolic composition.

Intracellularly, vesicle dynamics can be interfered through nanomagnetic forces localized on a magnetic cell culture platform (Figure 6E). This method makes use of internalized magnetic nanoparticles which are encapsulated in membrane-originated lipid vesicles (Figure 6H). Through the application of nanomagnetic forces on chip, (Figure 6F) the motility and transport direction of lipid vesicles in primary cortical neurons was either stalled or re-directed, even against insulin-mediated chemical signals (Figure 6I; Kunze et al., 2017). In addition to magnetic forces, optical tweezer platforms provide a similar force range as nanomagnetic forces and have been probed for transporting and positioning of recycling endosomes and perixomes and RAB11 vesicles in COS-7 cells and primary hippocampal neurons (Harterink et al., 2016). The specificity of positioning organelles with optical tweezers, however, requires the knowledge of expressing tunable, light-controlled interacting protein tags in cells of interests (Strickland et al., 2012).

## Magnetofection

Magnetofection is a transfection technique in which an external magnetic field is utilized to improve delivery of nucleic acids attached to MNPs into cells. This technique was originally conceived by Plank et al. (2003). Recently, a study conducted by Smolders *et al*. compared the efficiency of magnetofection to other transfection methods using a microglial cell line. They found that Glial-Mag magnetofection of BV2 cells greatly outperformed standard chemical transfection methods; calcium-phosphate precipitation, X-tremeGENE, and Lipofectamine 2000 with an efficiency of 34.95% compared to 0.34, 3.30, and 12.51% respectively (Smolders et al., 2018). In contrast to this study, however, Katebi et al. found that a static magnetic field reduces the uptake of exogenous oligonucleotide by rooster spermatozoa (Katebi et al., 2016). They observed that when primary spermatocytes were incubated in exogenous oligonucleotide solution with MNPs, the uptake was increased, however, when the static magnetic field was applied, a significant decrease in uptake occurred (Katebi et al., 2016). This indicates that the application of a static magnetic field may prove detrimental to different cell types and that further research should be conducted. Of particular interest would be what effect may the static field have on primary neuronal cells from different origins.

In contrast to static magnetofection, the method has been further developed to use oscillating magnetic fields. Fouriki et al. found that the application of an oscillatory field increased fluorescence intensity of transfected human embryonic kidney cells (H292) (Fouriki et al., 2010). This method has been applied, with frequency dependent efficiency, to rat astrocytes as well as neural stem cell in suspensions (Pickard and Chari, 2010; Adams et al., 2013). Adam et al. demonstrated a two-fold increase in transfection efficiency on neural stem cell suspensions at 4 Hz with no effect on cell viability, number, marker expression or differentiation profiles, indicating a safe transfection method for neural stem cells (Adams et al., 2013).

While the application of nanomagnetic forces has been well demonstrated in increasing transfection efficiency, further research needs to be done on the applications of oscillating magnetic fields. Current research is indicative of a frequency dependent component of oscillatory magnetofection that it may be possible to optimize. Furthermore, magnetically targeting specific individual cell types within a cell population is specifically interesting to study disease models *in vitro*. The spatial limitation of magnetofection, however, currently remains a challenge, because the externally applied macro magnetic fields will always impose a spatial magnetic gradient across the entire cell culture platform. The effect on other cells types within the same culture currently remains unknown. Thus, increasing spatial resolution and specificity of magnetic gradients down to single cell levels can be the focus of a variety of future studies.

## Future challenges and perspective

The purpose of this review was to highlight emerging applications of nanomagnetic forces and related concepts such as magnetic field effects and differences between permanent and alternating magnetic field stimulation on mammalian cell behavior. We have discussed several advantages of nanomagnetic force stimulation over other force-mediating methods, however, we do need to acknowledge that our current understanding of nanomagnetic force stimulation has its limits. While magnetic field gradient can penetrate tissues, organs, or the human body it currently remains challenging to operate nanomagnetic forces in a controlled and precise manner through three-dimensional tissue constructs. Furthermore, the response of cells to nanomagnetic force stimulation is limited in time. In the following, we would like to outline our opinion about how studies involving nanomagnetic force stimulation can address (i) spatiotemporal limitations of end-point experiments and (ii) bring this technology away from the bench and integrate it into mechanically-mediated diagnostics, pharmaceutical cell assays, and neurotherapeutics.

### Spatio-temporal response

Current studies about cell-based nanomagnetic force stimulation compare cell effects based on endpoint measurements or based on short time-windows of several minutes, as in the case of calcium stimulation. It means that our current knowledge about nanomagnetic force stimulation in biological systems stems from either several minutes of live-cell experiments, or few day endpoint experiments (24 h and more) without access to capture time-related intermediate data. From the endpoint measurements, we can conclude how nanomagnetic forces interfere with cells and which down- or upstreaming cell signals get activated or inhibited. How cells, however, adjust temporally over a period of days or months to a potential nanomagnetic-based treatment requires a better understanding of the spatial-temporal relation between the force stimulus and the cellular, tissue and organ response. Systematic long-term experiments, where cell growth and behavior are constantly monitored using either optic, or electric measurements without interfering with the experimental setting, would allow us to learn more about spatio-temporal response of nanomagnetic forces stimulation.

**Figure 7:**
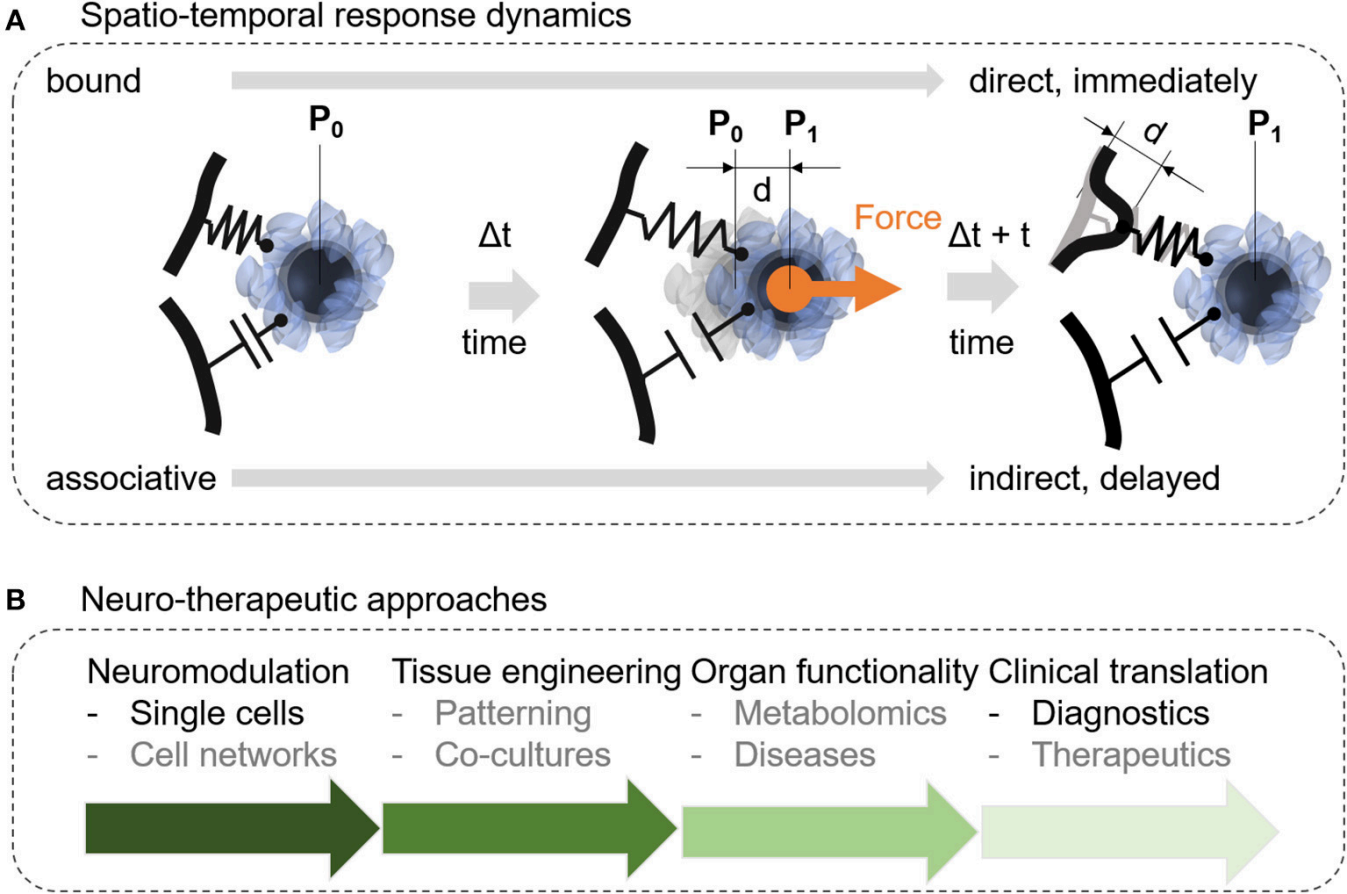
Suggested future studies should address fundamental aspects of how nanomagnetic forces associate with cellular structures or how nanomagnetic force stimulation can be integrated into therapeutic and translational approaches. **(A)** Protein-protein interactions are suggested to play a dominant role in nanomagnetic force activation and may determine how much force is required and how sensitive cells are to a biomechanical stimulus at the membrane. Depending on the surface functionality nanoparticles may interact with the cellular membrane in a weak associative or on a strong bound connection. The strong bound connection suggests an immediate deformation of the membrane resulting in a short lag time to trigger a specific intracellular downstream process after a stimulus occurred. In contrast to the strong connection, the weaker associative connection may lead to a longer lag time or result in no further activation of downstream processes. **(B)** Other research efforts should focus on integrating nanomagnetic force stimulation into current neuromodulation tools, tissue engineering, organ functionality and translation into diagnostics, patient-specific therapeutics, or treatment predictions.

Future nanomagnetic force-mediating studies may reveal new properties about the link between the force-mediating object and the cellular response. Figure 7A depicts two potential mechanism how the nanoparticle may translate the force stimulus to the cellular structure based on a direct or an associative link. The link between the nanomagnetic force stimulus and the subcellular object (organelle, cell membrane, cytoskeleton) impacts the time lag for the cellular response. If the nature of this link between the nanoparticle and the cellular structure is direct, the cellular response should be seen almost immediately. After a force stimulus, the cell would need to at least interpret this stimulus *in situ*, if not triggering downstream signals immediately. In contrast, an associative link contains a storing capacity. The unloading of this capacity may or may not follow within the same time lag as for the direct link. It is more likely, however, that the storing capacity of the associative link triggers a cellular response within minutes, hours, or days. Thus, the time-lag will be an important parameter to better understand mechano-transduction and translational approaches in nanomagnetic force stimulation. Furthermore, Ricca et al. suggests within the context of mechanotransduction to use clearly defined extracellular mechanical cues as input signals to elucidate between an active and a passive input (Ricca et al., 2013). Within the context of our bound or associative nanoparticle which can be controlled through engineered surface coatings, the passive input can be modeled through an associative link and would show a delayed cellular response in comparison to the active, bound link. Concerning neurotherapeutic approaches, this delayed effect will be either desired, controlled, or prevented. Therefore, a deeper understanding of the spatio-temporal aspects of nanomagnetic force stimulation is essential to prepare this approach for further translational studies.

### Neurotherapeutics

Our literature review focused on current single and multi-cell applications using nanomagnetic forces and related magnetic actuation concepts where we see a potential for translational applications regarding neurotherapeutics. In this last section, we want to provide to the reader an overview with the diverse potential of nanomagnetic force stimulation in translational research, neurotherapeutics and patient-specific prognostics (Figure 7B). In the previous section, we have outlined a fundamental question regarding the temporal response of nanomagnetic force stimulation, which needs to be answered for a variety of translational applications, nevertheless, we assume that this knowledge will be available in > 10 years. To truly realize the potential of nanomagnetic force stimulation, we need to go beyond single cell analysis and ask how nanomagnetic force stimulation will impact cell networks, specifically connected neuronal cell circuitries. The next step toward neurotherapeutics is to incorporate nanomagnetic force stimulation into neural tissue engineering, (Goldberg et al., 2007; Ito and Kamihira, 2011) into delivery mechanism of biopharmaceuticals across the blood brain barrier, (Thomsen et al., 2015) or into axon elongation strategies for repairing spinal cord injuries (Kilinc et al., 2016). The potential of adding magnetic force stimulation to tissue engineering lays in the properties of the nanoparticles to modulate cell mechanics (Septiadi et al., 2018) and to induce controlled forces within extracellular constructs to switch between different mechanical properties through turning on and off the magnetic field (Zhang et al., 2016). The latter approach is beneficial to squeeze drugs out from a scaffold for a controlled duration during mechanically-force triggered drug delivery (Zhang et al., 2016). The transport of biopharmaceuticals through the blood brain barrier can further be promoted through magnetic force applications in combination with magnetoliposomes (Thomsen et al., 2015). Adding nanomagnetic forces stimulation to neural grafts for spinal cord repair can be an alternative to optogenetic approaches (Bryson et al., 2016; Kilinc et al., 2016). Finally, the differential uptake of magnetic nanoparticle into different brain cell types can be used to either selectively target and sort specific brain cell types, or to build controlled patterns of brain cells for artificial neural tissues. Further translation of nanomagnetic force stimulation into brain issues and neurotherapeutics will also require a systematic understanding of brain cell functionality through metabolomics and proteomics (Holle et al., 2018). Last, magnetic nanoparticles, which are the core of nanomagnetic forces are already common in cell sorting for cancer-based diagnostics, however, there is plenty of room to come up with new methods to integrate nanomagnetic forces into mechanically-mediated diagnostics and neuro- therapeutics based on protein chaperoning, separation, and on-chip cell technology.

## Author contributions

TG: Reviewed literature and wrote parts of the review paper; AK: Supervised, organized, reviewed literature and wrote this review paper. Both authors revised the manuscript.

### Conflict of interest statement

The authors declare that the research was conducted in the absence of any commercial or financial relationships that could be construed as a potential conflict of interest.
